# High-Antimicrobial Gallium-Doped Zinc Oxide Thin Films on Bio-Based Poly(Ethylene Furanoate) Substrates for Food Packaging Application

**DOI:** 10.3390/membranes13020239

**Published:** 2023-02-17

**Authors:** Chaoting Zhu, Danling Ye, Tianqi Zhou, Yashuang Cui, Jianbing Yin

**Affiliations:** 1 Institute of Medical Instruments, Zhejiang Pharmaceutical College, Ningbo 315201, China; 2 Center for Medical Device Adverse Events Monitoring of Zhejiang, Zhejiang Medical Products Administration, Hangzhou 310009, China

**Keywords:** GZO thin films, poly(ethylene furanoate), antimicrobial properties

## Abstract

Thin films of gallium-doped zinc oxide (GZO), with a thickness of around fifty nanometers were deposited on bio-based poly(ethylene furanoate) (PEF) substrates by radio-frequency sputtering. By optimizing the Ga concentration in the target, the optics, water vapor barrier and antibacterial properties of PEF/GZO composite films can be adjusted. The highest visible light transmittance of the samples was around 85.1%. Furthermore, by introducing some GZO films with typical concentrations, the water vapor barrier and antibacterial properties of PEF films were improved. The optimized water vapor permeability of PEF/GZO composite film was 5.3 × 10^−12^ g·m/m^2^·s·Pa, and the highest antibacterial rate can reach 99.85% after 4 h. By XPS analysis, the antibacterial mechanism in the samples is envisaged to be mainly due cytotoxicity of Ga ions. The above results indicate that PEF/GZO films have great potential in the field of antibacterial food packaging.

## 1. Introduction

Currently, most of the single-use plastic products used worldwide are processed from non-renewable petroleum [1,2,3]. Environmental pollution caused by petroleum-based plastics is becoming increasingly serious, and food safety problems caused by traditional plastic packaging are prominent [4,5,6]. Therefore, vigorously promoting low carbon, green and renewable food packaging materials is the main way to solve the current food packaging problems [7,8].

Poly(ethylene furanoate) (PEF) is a recyclable bio-plastic with a chemical structure similar to poly(ethylene terephthalate) (PET) [9,10,11]. The raw material for PEF synthesis is composed of 2,5-furanediformic acid (FDCA) and ethylene glycol (EG), in which FDCA can be extracted from biomass [12,13]. The physicochemical properties of PEF are similar to those of PET, for example, the two have similar glass transition temperatures and dissolution characteristics [14]. As a packaging film, PEF has better water vapor barrier properties than PET, thus providing better freshness preservation [15]. Moreover, PEF has higher mechanical strength, which can produce thinner packaging, thereby reducing packaging weight of films and bottles [16].

In the field of food packaging, researchers often introduce antimicrobial agents (organic and inorganic antibacterial agents) into packaging films to improve the shelf life of the food inside [17,18,19]. Among them, inorganic antibacterial materials, such as Ag, TiO_2_, ZnO, etc., are often added to the preparation process of antibacterial packaging films [20,21,22]. Compared with organic antibacterial agents, inorganic antibacterial agents exhibit stronger environmental stability, both in high-temperature and high-pressure environments during plastic molding processing and in daily use, and they generally show strong antimicrobial activity even in small amounts [22].

Recently, ZnO nanoparticles (ZnO NPs) have been introduced into food packaging materials due to their abundant reserves, low cost and inhibitory effect on the propagation of *Escherichia coli* (*E. coli*) and *Staphylococcus aureus* [23,24]. Bulk ZnO has been generally recognized as safe (GRAS) by the US Food and Drug Administration (FDA) (21CFR182.8991) but there is less certainty regarding nano-sized ZnO because of their size, which is of the same dimensions as biomolecules such as DNA, enzymes, proteins present in living cells [25]. The potential toxicity of ZnO NPs to humans remains controversial [26]. Nanoparticle migration and release of composite packaging films remain to be investigated [27]. 

Compared to the incorporation of ZnO NPs into the film, the deposition of ZnO-based antibacterial coatings on the film surface by physical vapor deposition (PVD) is an alternative option, similar to aluminum foil packaging. The coating mode of PVD not only ensures the good adhesion of ZnO films to the substrate but also avoids the generation of ZnO NPs. However, compared with the high antibacterial property of ZnO nanoparticles, the antibacterial property of pure ZnO films prepared by the PVD method has a significant decrease due to the reduced specific surface area. Therefore, aluminum, copper and other metal antibacterial elements were introduced into ZnO films to improve their antibacterial activities. Recently, D.Valini et al. have used magnetron sputtering to prepare PLA/AZO composite films with antibacterial properties, achieving 99% antibacterial efficiency against *Escherichia coli* [28]. However, the solid solubility of aluminum elements in ZnO films is low, and transition doping will lead to a decrease in its visible light transmittance, which will affect the packaging use. A similar phenomenon has been reported in the study of copper doped ZnO films by Iman A. Hassan et al. [29]. In contrast, gallium-doped ZnO (GZO) films have a high effective doping content due to the close Ga-O (1.92A) and Zn-O (1.97A) bond lengths. In our previous study, we found that when the gallium doping concentration is 6 wt%, the 500 nm GZO film still has a visible light transmittance of about 82% [30]. More importantly, low levels of gallium are almost non-toxic to the human body and have important applications in clinical disease diagnosis and treatment. For example, the radioactive isotope ^67^Ga captures electrons in the decay process and releases gamma rays, which is used for staging and diagnosis of clinical lymphoma [31]. ^68^Ga can release positron and is used as a contrast agent for clinical positron tomography imaging [32]. Gallium nitrate is also used in the treatment of clinical cancer-related hypercalcemia, which is the first Ga compound approved for clinical use [33]. The gallium-containing substances mentioned above did not show obvious toxic side effects in the course of treatment. In addition, gallium elemental antibacterial agents belong to the category of metal antibacterial agents, which have broad spectrum antibacterial properties and excellent antibacterial properties without drug resistance [34]. In conclusion, GZO/PEF composite film may be a potential new antibacterial packaging transparent bio-based film. Therefore, we attempted to fabricate ZnO coatings with different gallium doping concentrations on the surface of PEF films and investigated their antibacterial, optical and water vapor barrier properties.

## 2. Experimental

### 2.1. Materials

PEF raw material was purchased from Dagott Technology Co., Ltd., Shenzhen, China. The matching phenol (C_6_H_5_OH) and methylene chloride (C_2_H_2_Cl_4_) were purchased from Runhui Biotechnology Co., Ltd., Guangzhou, China. The GZO ceramic target for sputtering was purchased from Beijing Jingmaiyan Material Technology Co., Ltd., Beijing, China.

### 2.2. Fabrication of PEF/GZO Composite Thin Films

The preparation process of PEF films was as follows: (1) mixing C_6_H_5_OH and C_2_H_2_Cl_4_ at a ratio of 1:1 as solvent; (2) According to the ratio of PEF raw material and solvent 1:10, the PEF solution was prepared by dissolving at 90 °C; (3) A certain proportion of PEF solution was placed on the surface of flat glass by drop coating method and placed in a 90 °C vacuum constant temperature drying oven for 2 to 3 h to remove the solvent; (4) The PEF film with flat glass should be left in deionized water for 2 to 3 h to facilitate the PEF film to be detached from the glass surface. (5) The obtained PEF film was put into an ordinary oven at 60 °C to dry until use. To ensure the uniformity of the subsequent PEF films, the size of the glass substrate used in this experiment was 10 cm × 10 cm, and the thickness of the film (~90 μm) was controlled by the volume of the pipetted gun. The specific PEF sample size can be tailored according to the test requirements.

The surface of the PEF film in contact with the flat glass was used as the substrate, and the GZO film of about 50 nm was deposited on the substrate by radio-frequency (RF) magnetron sputtering (VTC-600-2HD, Shenyang Kejing, Shenyang, China). The specific conditions of GZO film deposition were as follows: sputtering power was 50 W, sputtering temperature was room temperature, pre-vacuum of cavity was 1 × 10^−3^ pa, sputtering gas is 25 SCCM argon, gallium concentration of target used for sputtering was 2 wt%, 3 wt%, 4 wt%, 5 wt% and 6 wt%, respectively. The PEF/GZO composite films were designated as G2O0, G3O0, G4O0, G5O0 and G6O0 according to the concentration of gallium in the target, respectively.

### 2.3. Films Characterization

The optical properties were measured by a UV-Vis spectrophotometer (Gold S54T, Lengguang, Shanghai, China) in the test band of 190 nm to 1100 nm. The optical band gap of the sample was obtained by using the Tauc method to calculate UV-vis data. The surface wettability of the samples was examined using an optical contact angle measuring instrument (OCA20, Data physics, Filderstadt, Germany). The surface morphologies of the samples were obtained by using a scanning probe microscopy (Dimension Icon, Bruker, Billerica, MA, USA). The thickness of the GZO film was measured by optical ellipsometer (ME-Mapping-L, Yiguang Technology, Shenzhen, China). The elemental chemical states on the surface of the samples were determined by a Kratos Axis Supra X-ray photoelectron spectroscopy (XPS) (AXIS SUPRA, Shimadzu, Kyoto, Japan) using monochromatic Al-Kα radiation. The elemental peaks were fitted using CasaXPS software (Casa Software Ltd., Qormi, Malta) and the binding energy was adjusted to C1s (284.8 eV) for charge correction. The tensile tests were performed with an electronic universal testing machine (ETM150D, Shenzhen Wantest, Shenzhen, China) at room temperature with a speed of 10 mm/min.

The water vapor transmission (WVT) and water vapor transmittance (WVP) of the samples were determined using a moisture permeability test system (XZ-C6, New quasi, Jinan, China). The test principle is the wet-permeable cup weighing method. The temperature and humidity set for testing were 38 °C and 89% RH, respectively. The test interval of the cup weight was set to 3 h, and the total test time was 24 h. Air permeability (VAc-V2, Labthink, Jinan, China) was tested using a differential pressure gas penetrator. CO_2_ and O_2_ were used as gas sources with pressures of 0.5 MPa, respectively. The temperature and humidity tested were 30 °C and 50% RH, respectively.

The antibacterial effect of the samples and PEF/ZnO films against *E. coli* (ATCC 29522) was determined by the membrane adhesion method. PEF/ZnO films were prepared using the same sputtering conditions described above. In addition, pure PEF films were used as the control group in this antibacterial experiment. According to ISO 22196-2011, the procedure was as follows: (1) Cover the sample (4 cm × 4 cm) on a Petri dish with 100 μL of *E. coli* suspension (~10^5^ CFU/mL); (2) The samples were placed in a 37 °C incubator and incubated for 4 h (sterilization time); (3) The sample was rinsed with 10 mL normal saline (0.2% tween 80) and continuously diluted with 10× normal saline for counting viable bacteria; (4) One ml of each diluent was placed into a sterile Petri dish, and an amount of nutrient AGAR (15–20 mL) was poured into each dish. After AGAR coagulation, the plates were incubated at 37 °C for 19 h; (5) The counts were performed in Petri dishes containing 30 to 300 colonies.

To further quantify the antibacterial effect, the formula for calculating the antibacterial rate was prescribed as follows:

(1) $$R\left(\%\right)=\frac{\left(B-C\right)}{B}\times 100\%$$

where R represents the antibacterial rate (%); B represents the average number of bacteria recovered from pure PEF samples (CFU/mL); C represents the average number of bacteria recovered from PEF/GZO and PEF/ZnO samples (CFU/mL). 

## 3. Results and Discussion

### 3.1. Optical and Surface Properties

**Figure 1:**
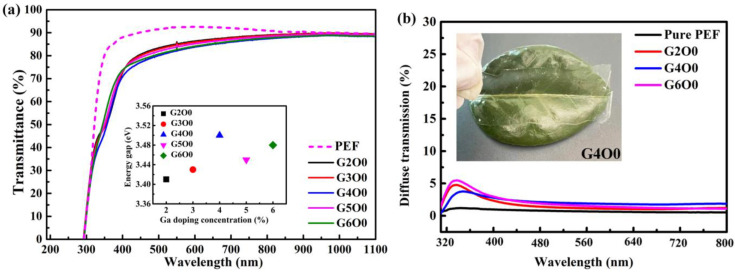
(**a**) Optical transmittance of PEF/GZO thin films as a function of Ga concentration in the target. The inset shows the optical band gap of the PEF/GZO film as a function of Ga concentration; (**b**) Diffuse transmittance of PEF thin film and PEF/GZO thin films (G2O0, G4O0, G6O0). The inset shows the photograph of the G4O0 sample.

Visible light transmittance is an important reference index to evaluate the optical properties of packaging films. Figure 1a illustrates the optical transmittance of PEF and PEF/GZO composite films. In the ultraviolet band, the transmission curve of the composite film shows an obvious decline due to the ultraviolet absorption characteristics of GZO film. In the visible region (400–800 nm), the average transmittance of all films exceeds 80%. The G2O0 sample has the highest average visible light transmittance of 85.1% in all GZO/PEF samples, which is 2.8% lower than that of pure PEF film. The decrease in transmittance is mainly attributed to the increase of the interface of the sample caused by the introduction of GZO film, which leads to the increase of the reflection. More importantly, the large difference in refractive index between ZnO and PEF materials further strengthens the reflection phenomenon between the two interfaces. With the increase of gallium doping concentration (2–4 wt%), the visible light transmittance of the corresponding composite films decreased slightly (from 85.1 to 82.6%). A similar phenomenon also occurred in the case of Cu doped ZnO films in reference [29]. In order to study the reason of transmittance decrease, the optical band gap width of all samples was fitted. The illustration in Figure 1a shows the band gap of GZO film as a function of Ga doping concentration. The band gap of GZO films increases with the Ga doping concentration in the range of 2–4 wt%. According to the Burstein–Moss effect, the band gap of the N-type semiconductor is closely related to its carrier concentration [35]. Therefore, the variation of band in the illustration represents the change of carrier concentration in GZO film. By further analysis of the transmission curve in Figure 1a, it can be found that the transmittance of the composite film had an opposite trend to the carrier concentration in the corresponding GZO film. Combined with the optical band gap results, it is not difficult to find that the carrier concentration in the sample actually shows a rising trend. This causes the absorption in the GZO film to begin to increase, which results in a decrease in the overall transmittance of the film. This explains that the composite film has the lowest visible light transmittance (82.6%) when the gallium doping concentration is 4 wt%. However, the visible light transmittance of GZO/PEF samples increased to a certain extent (82.6–83.1%), when the gallium doping concentration increased from 4 wt% to 6 wt%. In particular, the G5O0 sample showed a visible light transmittance of 84.1%. When the doped concentration of gallium increased further, the band gap began to decrease due to the limitation of solid solubility. Too much gallium doping does not effectively displace Zn atoms in the ZnO lattice to produce effective charge carriers. The excess gallium element can only exist in the film in the form of gap gallium or gallium oxide, which leads to ineffective doping. This results in a decrease in absorption in the GZO film, which increases the transmittance. 

Another evaluation of optical properties of packaging films is visible light scattering. Too much light scattering can lead to a fog-like appearance of the film. To this end, we tested the scattered transmitted light of typical samples, and the results are shown in Figure 1b. It can be found that compared with the pure PEF film, the GZO/PEF film has a certain scattering phenomenon, but it is not obvious in general. The inset in Figure 1b is the physical picture of G4O0, from which it can also be seen that its light scattering scene is not serious.

**Figure 2:**
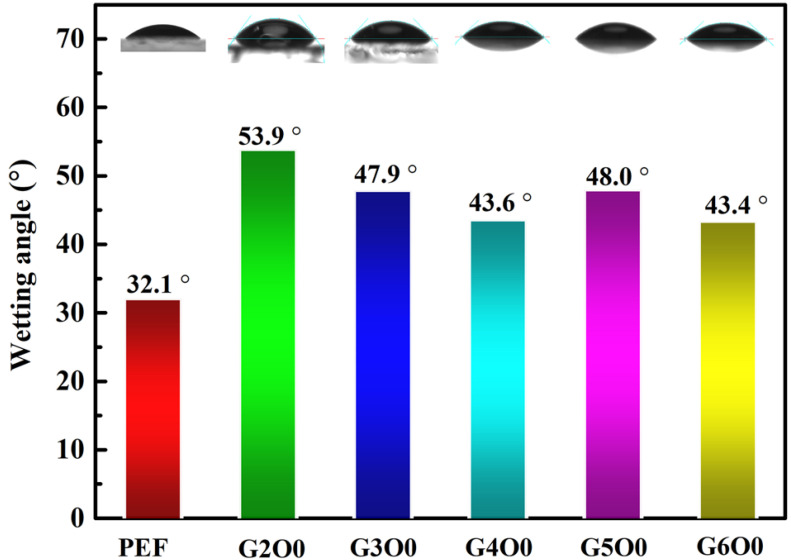
Contact Angle test results for PEF/GZO films as a function of Ga concentration in the target.

Bacterial adhesion on the surface of packaging films is often the beginning of biofilm formation. Understanding and controlling bacterial adhesion is of great significance for the application of packaging films. However, bacterial adhesion is a complex process controlled by many factors, including the structural composition of bacterial cells, the surface properties of adsorbed objects, and the characteristics of suspensions. Among them, the infiltration of the membrane surface is particularly important to the effect of bacterial adhesion. The wetting angles of pure PEF film and PEF/GZO composite films were measured at room temperature using a contact angle measuring instrument (Figure 2). The results show that the contact angle of pure PEF film was 32.1°, showing obvious hydrophilicity. Generally speaking, hydrophilic surfaces are not conducive to the attachment and growth of microorganisms and bacteria [36]. However, after the deposition of GZO film, the contact angle of the G2O0 sample (the wetting angle: 53.9°) is obviously improved due to the change of surface characteristics. This change is not friendly to the antibacterial properties of the packaging film. For this purpose, we further analyzed the contact angle test results of samples with different gallium doping concentrations. Figure 2 shows that the contact angle of the PEF/GZO composite film decreases from the initial 53.9° to 43.6° as the gallium doping concentration increases from 2 wt% to 4 wt%. On the other hand, when the gallium doping concentration is in the range of 4 wt% to 6 wt%, the wetting angle of the composite film is maintained in the range of 43.4° to 48.0°. 

**Figure 3:**
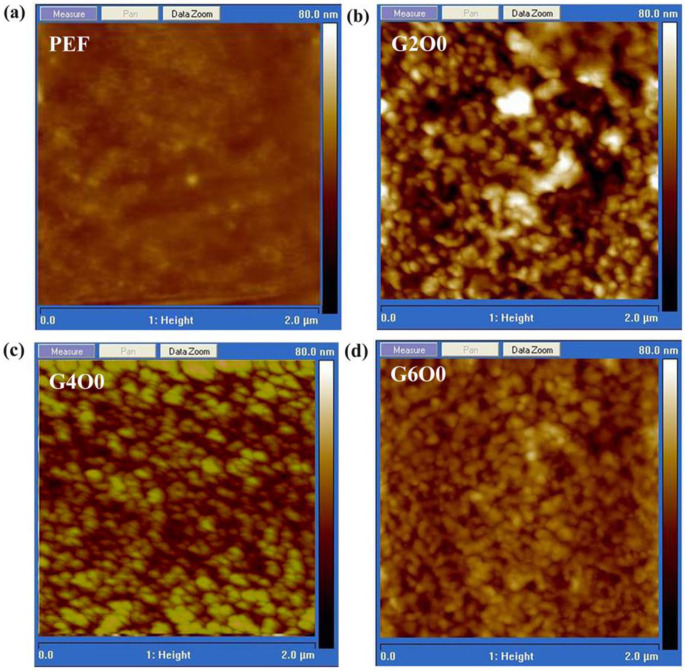
AFM images of PEF (**a**), G2O0 (**b**), G4O0 (**c**) and G6O0 (**d**).

A large number of studies have shown that the wettability of films is closely related to the surface chemical composition and roughness [37,38]. In order to explore the reasons for the variation of the contact angle, the surface roughness of some samples was measured using scanning probe microscopy, and the results are shown in Figure 3. The rms roughness of pure PEF is 1.81 nm, showing excellent flatness (Figure 3a). Distinct the high flatness of pure PEF film, the rms roughness of G2O0 sample is significantly improved to 7.55 nm (Figure 3b). For the G4O0 and G6O0 samples, their rms roughness is extremely close, and the values are 4.76 nm and 4.72 nm, respectively (Figure 3c,d). Taking into account Wenzel’s equation:

(2) $$\cos\theta^{*}=\gamma \cdot \cos\theta =\gamma \cdot \frac{\varphi_{sv}-\varphi_{sl}}{\varphi_{lv}}$$

where $\theta^{*}$ is the measured contact angle of a sample, θ is Young’s contact angle, γ is the rough factor, $\varphi_{sv}$ is solid–vapor interfacial energy, $\varphi_{sl}$ is solid–liquid interfacial energy, $\varphi_{lv}$ is liquid-vapor interfacial energy, when the sample surface is in a hydrophilic state (θ < 90°), an increase in roughness (γ) will lead to a decrease in the sample contact angle ($\theta^{*}$). This interpretation is completely opposite to the results of the sample contact angles measured. This indicates that the surface roughness of the sample is not the main factor affecting its surface wettability in this study. Correspondingly, the chemical bonds on the sample surface change the solid–liquid and solid–gas interface states, thus affecting the final contact angle test results. For example, when GZO film is deposited on the surface of PEF film, the contact angle of the composite film is significantly increased due to the lower surface free energy of ZnO film. When the gallium element is added to the ZnO film, the presence of Ga_2_O_3_ on the surface brings more OH groups, which leads to a more hydrophilic ZnO surface. A similar phenomenon also occurs in the studies of Cu-doped ZnO films, as in references [29,36].

### 3.2. Water Vapor and Gas Barrier Properties

**Figure 4:**
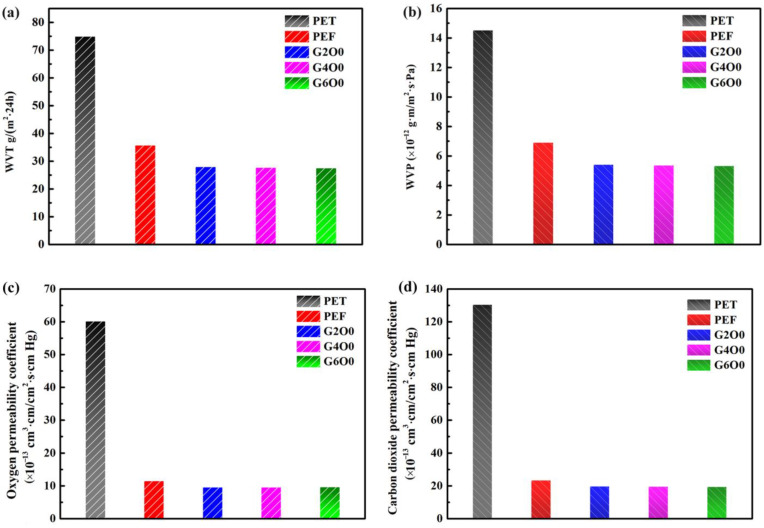
(**a**) Water vapor transmission of PET, PEF, G2O0, G4O0 and G6O0. (**b**) Water vapor permeability of PET, PEF, G2O0, G4O0 and G6O0. Oxygen permeability coefficient (**c**) and Carbon dioxide permeability coefficient (**d**) of PET, PEF, G2O0, G4O0 and G6O0.

Water content and activity in food is one of the main factors affecting food quality and stability during storage [39,40]. Therefore, the good moisture barrier effect of packaging film is of great significance to prolong the shelf life of food. Figure 4a,b show the WVT and WVP values of pure PEF and PEF/GZO composite films. In order to be more intuitive, the same conditions were used to test the moisture permeability of commercial PET films (~90 μm). The WVT and WVP values of PET films were 74.9 g/m^2^·24 h and 1.45 × 10^−11^ g·m/m^2^·s·Pa, respectively. For the pure PEF film, the WVT and WVP values both decreased by about half, to 35.7 g/m^2^·24 h and 6.9 × 10^−12^ g·m/m^2^·s·Pa, respectively. These data indicate that PEF has a significant improvement in the water-vapor barrier compared to PET films. In order to improve the barrier of the packaging film, vacuum coating is an alternative method. In this study, GZO films (~50 nm) with various Ga doping concentrations were deposited on the surface of PEF films. Three samples with typical gallium concentrations (G2O0, G4O0 and G6O0) were selected for testing, and the results showed that the water-vapor barrier of the corresponding PEF/GZO composite films was slightly improved, with WVT values in the range of 27.4 g/m^2^·24 h and 27.9 g/m^2^·24 h, and WVP values in the range of 5.3 × 10^−12^ g·m/m^2^·s·Pa and 5.4 × 10^−12^ g·m/m^2^·s·Pa. In addition, we tested the oxygen and carbon dioxide gas barrier properties of the samples. The test results are shown in Figure 4c,d. The oxygen permeability coefficient and carbon dioxide permeability coefficient of PET were 6.01 × 10^−12^ cm^3^·cm/cm^2^·s·cm Hg and 1.3 × 10^−11^ cm^3^·cm/cm^2^·s·cm Hg, respectively. The gas permeability coefficient of PET was denoted as 1. Correspondingly, the oxygen permeability coefficients of PEF, G2O0, G4O0 and G6O0 were 5.27, 6.33, 6.34 and 6.29 times lower than those of PET. The carbon dioxide permeability coefficients of PEF, G2O0, G4O0 and G6O0 were 5.58, 6.59, 6.66 and 6.69 times lower than those of PET. These results indicate that the improved barrier properties of PEF/GZO composite films are independent of the Ga doping concentration in the GZO films and may be related to factors such as the density and thickness of the GZO films. 

### 3.3. Antimicrobial Properties

**Figure 5:**
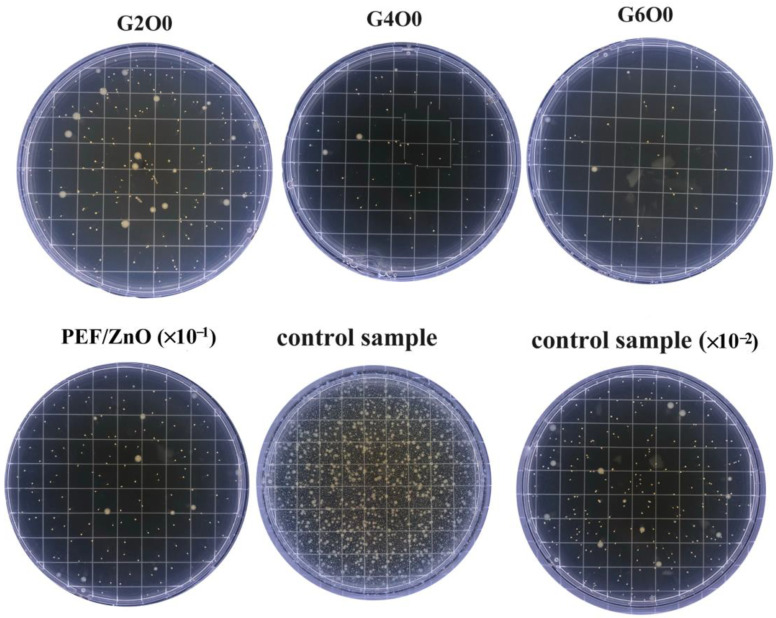
Antimicrobial activity of PEF, PEF/ZnO, G2O0, G4O0 and G6O0 against *E. coli* for 4 h.

Coating the surface of packaging with inorganic antibacterial agents is a greener way to fight bacteria than adding preservatives to the packaging [41,42]. The antimicrobial activity of pure PEF films (control sample), PEF/ZnO films and PEF/GZO composite films were measured using a common gram-negative bacterium *E. coli*. Figure 5 illustrates the growth of *E. coli* in the typical samples after 4h of antimicrobial treatment. It can be seen that the number of *E. coli* colonies in the G2O0, G4O0 and G6O0 samples were greatly reduced compared with the pure PEF film (control sample). In addition, with the increase of Ga doping concentration in the GZO thin films, the number of visible colonies in the samples also decreased significantly. The detailed colony numbers of *E. coli* in PEF/ZnO film, G2O0, G4O0 and G6O0 were 1810 CFU/mL, 252 CFU/mL, 69 CFU/mL and 46 CFU/mL, respectively (Table 1). The corresponding antibacterial rates of PEF/ZnO film, G2O0, G4O0 and G6O0 against *E. coli* for 4 h reached 94.14%, 99.18%, 99.78 and 99.85%, respectively. The above results indicate that the introduction of GZO coating makes PEF films have a certain antibacterial property, and the antibacterial property of the composite films increases with the increase of Ga concentration in the GZO films.

**Figure 6:**
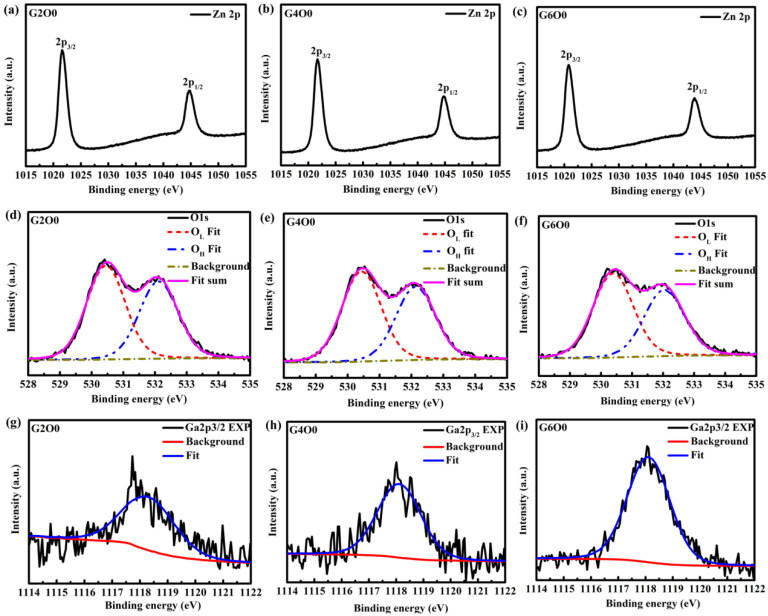
High resolution XPS spectra of Ga, Zn and O in GZO thin films: (**a**) Zn 2p of G2O0, (**b**) Zn 2p of G4O0, (**c**) Zn 2p of G6O0, (**d**) O 1s of G2O0, (**e**) O 1s of G4O0, (**f**) O 1s of G6O0, (**g**) Ga 2p_3/2_ of G2O0, (**h**) Ga 2p_3/2_ of G4O0, and (**i**) Ga 2p_3/2_ of G6O0.

In order to further analyze the reasons for the improvement of antibacterial activity of composite films, GZO films with the same conditions were prepared on glass substrates for XPS test. The XPS results are shown in Table 2 and Table 3 and Figure 6. From Figure 6a–c, it can be found that the 2p peaks of zinc in typical samples (G2O0, G4O0 and G6O0) are symmetrical, indicating that zinc has only one valence state [43]. In addition, since the peak of Zn 2p_3/2_ is around 1021 eV (see Table 2), it can be inferred that the zinc in the sample is in the valence state of Zn^2+^. The O1s peaks (Figure 6d–f) are asymmetrical, indicating the presence of more than one oxygen species in the near-surface region of the GZO sample. According to the obtained spectral shapes, the O 1s XPS spectra of GZO samples are fitted into two peaks. The peak at about 530 eV in samples is closely related to lattice oxygen (O_L_), and the peak at about 532 eV is related to oxygen in the surface hydroxyl group (O_H_). Figure 6g–i show the fitting results of Ga 2p_3/2_, whose peaks are all around 1118 eV, indicating that gallium is mainly present in the form of Ga_2_O_3_ [44]. Moreover, it is not difficult to find that the intensity of Ga 2p_3/2_ peak increases with the concentration of Ga in the target. To further quantify the results of XPS analysis, the atomic percentages of Zn_2p_, O_L_, O_H_ and Ga_2p_ and the relative ratios of O_L_/(Zn_2p_ + Ga_2p_) were obtained according to the calculation of peak area and atomic sensitivity factor, which were summarized in Table 3. By analyzing the data in Table 3, it is easy to find that gallium content in GZO thin films increases significantly, from 0.98% to 2.65%. Correspondingly, the Zn content in the film decreased. In addition, the oxygen vacancy (V_o_) concentration in the surface layer of the sample does not change significantly. Generally, the antibacterial activity of ZnO films is mainly controlled by reactive oxygen species (ROS) and release of zinc ions (Zn^2+^) [45,46]. The former kills bacteria by generating ROS, such as ⋅O_2_^−^ and ⋅OH, through photocatalysis and V_o_ in the film. The latter kills bacteria through the cytotoxicity of heavy metal ions. In this study, ROS production mainly came from oxygen vacancies in GZO films because the antibacterial process of the sample was under dark conditions. Based on the analysis of V_o_ in XPS, it can be basically ruled out that ROS is the main factor changing the antibacterial property of the samples. Further analysis of changes in the content of Ga and Zn in the film, it can be concluded that the release of Ga^3+^ is probably the main mechanism of improving the antibacterial activity of GZO thin films.

### 3.4. Mechanical Properties

As a kind of packaging film, mechanical properties are also a very important performance index. Table 4 enumerates the test results of the mechanical properties of PET, PEF and PEF/GZO samples. The tensile strength of all PEF samples ranged from 83 to 87 Mpa, an improvement of 37% over PET samples. In addition, the Young’s modulus of the PEF sample was improved by about 49.2% compared to the PET sample. However, the elongation at break of PEF samples is only about 5%, which is much lower than that of PET samples. The main reason for these phenomena is that the furan ring chain structure in PEF is more rigid than the benzene ring chain structure in PET. 

## 4. Conclusions

In summary, GZO films with different doping concentrations were successfully deposited on PEF substrates by RF magnetron sputtering. When the Ga concentration in the target was 2 wt%, the PEF/GZO composite film exhibited the highest visible light transmittivity, up to 85.1%. By introducing GZO film, the water vapor barrier of PEF film was improved, and the WVP value was up to 5.3 × 10^−12^ g·m/m^2^·s·Pa. In addition, with the increase of Ga concentration in the target, the antibacterial activity of the composite film was significantly improved, and the highest antibacterial rate of 99.85% was obtained when 6 wt% Ga concentration in the target. By analyzing the XPS results, it is envisagedthat the reason for the improved antibacterial property of the composite films was the enhanced cytotoxicity caused by the increased Ga content in the GZO films. In addition, the mechanical properties of PEF/GZO samples and PET samples were compared. From the test results, it can be concluded that PEF/GZO films have stronger tensile strength and elastic modulus. Therefore, PEF/GZO films were expected to be used in the field of food packaging as a new bio-based antibacterial material.

## Figures and Tables

**Table 1:** Antimicrobial activity of PEF, PEF/ZnO, G2O0, G4O0 and G6O0 against *E. coli* for 4 h.

Samples	Number of Colonies in the Eluent (CFU/mL)	Bacteriostatic Rate/%
PEF/ZnO G2O0	1810 252	94.14 99.18
G4O0	69	99.78
G6O0	46	99.85
PEF (control)	3.09 × 10^4^	~

**Table 2:** Position of photoelectron lines of Zn 2p_3/2_, O 1s, and Ga 2p_3/2_ from the G2O0, G4O0 and G6O0 samples.

Samples	O 1s		Zn2p_3/2_ (eV)	Ga 2p_3/2_ (eV)
	O_L_ (eV)	O_H_ (eV)		
G2O0	530.4	532.1	1021.7	1118.3
G4O0	530.5	532.1	1021.8	1118.2
G6O0	530.4	532.0	1021.9	1118.1

**Table 3:** Atomic composition and Oxygen vacancy (Vo) of the G2O0, G4O0 and G6O0 samples.

Samples	Percent of Zn2p	Percent of O_L_	Percent of O_H_	Percent of Ga2p	Atomic Ratio of	V_O_ (%)
	(%)	(%)	(%)	(%)	O_L_/(Zn2p + Ga2p)	
G2O0	40.64%	32.01%	26.37%	0.98%	0.769	23.1%
G4O0	40.16%	32.32%	25.58%	1.94%	0.768	23.2%
G6O0	39.65%	32.23%	25.47%	2.65%	0.762	23.8%

**Table 4:** Mechanical properties of PET, PEF, G2O0, G4O0 and G6O0.

Sample	Tensile Strength (M Pa)	Young’s Modulus (G Pa)	Breakage Elongation (%)
PET	62	1.91	108
PEF	83	2.85	5.1
G2O0	86	2.91	4.9
G4O0	84	2.87	5.0
G6O0	87	2.92	5.0

## Data Availability

The data that support the findings of this study are available from the corresponding author upon reasonable request.
